# Mapping Shade Availability and Use in Zoo Environments: A Tool for Evaluating Thermal Comfort

**DOI:** 10.3390/ani10071189

**Published:** 2020-07-14

**Authors:** Jason D. Wark, Natasha K. Wierzal, Katherine A. Cronin

**Affiliations:** Animal Welfare Science Program, Lincoln Park Zoo, Chicago, IL 60614, USA; tkwierz@gmail.com (N.K.W.); kcronin@lpzoo.org (K.A.C.)

**Keywords:** zoo, shade, temperature, behavior monitoring, behavioral thermoregulation, ZooMonitor, Sichuan takin

## Abstract

**Simple Summary:**

The thermal environment experienced by animals in zoos has implications for their comfort, and ultimately welfare. In this study, we describe a simple low-cost approach to documenting one key aspect of the environment, shade, and its use by animals. We share a successive approach that can be adopted based on an organization’s capacity: Ranging from simple mapping of shade availability in enclosures that can be built upon to incorporate more advanced tracking of space use by animals and detailed assessment of shade use. Using these methods, we discovered shade availability at a zoo in a northern continental climate varied greatly across enclosures, as well as by season and time of day. We present a case study on Sichuan takin to highlight the applied potential of this approach. Relying on insights from a combination of shade and behavioral data, a shade structure was installed and then evaluated for this species. As zoos seek to create enclosures that promote positive welfare, careful consideration should be paid to the thermal environment and choices available to animals. We share these accessible methods to encourage others to evaluate shade and its use by zoo animals.

**Abstract:**

For many species in zoos, particularly megafauna vulnerable to heat stress, shade is a key environmental resource. However, shade availability has received comparatively less attention than other aspects of the zoo environment. In this study, we share a simple low-cost approach that we applied to document shade availability across 33 zoo enclosures. We then combined these assessments with behavioral observations of enclosure use and shade-seeking behavior during summer months in a case study focused on Sichuan takin (*Budorcas taxicolor tibetana*) (*n* = 3), a large cold-adapted bovid. Behavioral observations were conducted before and after installation of a shade sail for the takin. Results indicated that shade availability varied widely across zoo enclosures, with the percent of shaded space ranging from 85 % to 22 % across enclosures during summer months. Shade was a dynamic resource and increased throughout the year and fluctuated across the day, with the least shade available midday. Takin showed general preferences for shaded areas near the walls of their enclosure but were observed using newly available shade from the shade sail after its installation. These accessible methods can be easily applied to assess shade within existing enclosures, evaluate enclosure modifications, and provide guidance for the design of new enclosures.

## 1. Introduction

Modern zoos are interested in creating environments that can enhance the welfare of individual animals. Previous studies have documented a range of environmental variables that can impact welfare, including noise, lighting, and human presence (e.g., [1,2,3,4,5,6,7]). The thermal environment is likely to play a key role in the behavior and welfare of individuals as well, particularly for megafauna that are commonly housed outdoors, given the metabolic consequences of a large body size and allometric scaling of surface area to volume [8,9]. With projected increases of global temperatures resulting from climate change, understanding the impact of the thermal environment on animals is now more important than ever [10]. Although the thermal environment and its impact has been extensively studied in agriculture [11,12,13,14] and laboratory [15,16] environments, relatively few studies have directly explored this dimension of zoo environments.

Studies of thermal comfort in people and animals have traditionally been derived from thermophysiological models. These models predict body temperature by describing physiological responses, such as metabolic changes, sweating, and shivering, in response to environmental conditions [17]. Building from these models, the concept of the thermoneutral zone has been used to describe the range of temperatures at which an animal can maintain a stable core body temperature without requiring metabolic changes or evaporative heat loss [18]. Thermoneutral zones have been widely used in animal studies as a proxy for thermal comfort [19,20,21], but, as Kigma et al. have noted, the thermoneutral zone and thermal comfort zone are not synonymous [22]. Thermal comfort incorporates the psychological perception of environmental conditions and will typically represent a narrower range of temperatures than the broader thermoneutral [22,23]. Thermal comfort models have traditionally assumed a passive response by individuals and steady-state environmental conditions [24]. More recent advances in understanding thermal comfort have highlighted the importance of considering real-world scenarios, including behavioral changes initiated by the animal [23]. 

Many behavioral strategies for maximizing thermal comfort have been described in animals, including adjusting activity, posture, and social proximity [25,26]. One primary response to thermal discomfort observed in many species is seeking out suitable microclimates. For example, chimpanzees living in savannah environments have been observed to utilize caves to avoid heat during the warmest times of the year [27]. Numerous studies in agricultural settings have documented the use of shaded locations for species housed outdoors [28,29,30]. As research accumulates demonstrating the important role of providing animals with choice and control in their environment [31], it is important to evaluate the availability of thermoregulatory choice and control to zoo-housed animals, and how the animals are in turn utilizing those options. 

Relatively few studies have explored the choices in the thermal environment available to zoo animals and their behavioral response to thermal stress. Langman et al. provided the most detailed studies evaluating the microclimatic conditions in zoo enclosures to date [32,33]. Using specialized equipment (hydrothermographs and pyrheliometers), these authors described the cooling benefits of shaded areas, particularly for reducing shortwave radiation. Although valuable in understanding the thermal environment in zoos, these studies did not explore the behavioral response of animals that may provide additional insight into thermal comfort. Combining these approaches, Wark et al. described microclimatic conditions and the behavioral response of zoo-housed black-and-white colobus monkeys [34]. In this study, the space use of the monkeys was strongly influenced by temperatures and preferential use of shaded areas was the primary behavioral thermoregulation strategy observed. Interestingly, the monkeys began transitioning to shaded areas at lower temperatures than expected by the upper critical temperature of the thermoneutral zone. This finding highlights the importance of acknowledging the additional psychological dimension of thermal comfort and the importance of considering the behavioral response of animals when evaluating animal welfare. 

In the present study, we describe methods for documenting shade availability within and between zoo enclosures, and measuring corresponding shade use by animals. In the first part of this study, we pay particular attention to how shade changes across seasons and time of day, as the environment in a temperate region is dynamic and a single-point assessment (i.e., steady-state assumption) would fail to consider this variability. In addition, we present a case study describing the use of shade by a group of Sichuan takin (*Budorcas taxicolor tibetana*), large bovids native to mountainous regions of China and Tibet. This case study evaluated the impact of installing a shade sail in the takin enclosure to increase shade, an enclosure modification initiated based on the results of the shade mapping data from part 1 of this study. To our knowledge, this is the first report of the behavioral thermoregulatory response of takin to ex situ environmental conditions. We present these methods as an accessible approach to evaluating the thermal environment of zoo enclosures and to assess the corresponding behavioral responses of animals. Understanding the impact of environment characteristics on behavior and space use by animals can inform the evaluation and future design of zoo enclosures and enhance welfare.

## 2. Materials and Methods

### 2.1. Part 1 Subjects: Outdoor Enclosures

Part 1 of this study focused on 33 outdoor enclosures at Lincoln Park Zoo in Chicago, IL, which included 144 animals of 30 different species (Table 1). Maps that would be used to record the presence of shade were made for each enclosure using the free online program Pixlr [35] Maps were made by updating the original design blueprints to match current enclosure features, resulting in a top-down “bird’s eye view” of the space. Only outdoor space was included in the maps (i.e., off-exhibit holding spaces were not included).

### 2.2. Part 2 Subjects: Animals 

**Figure 1:**
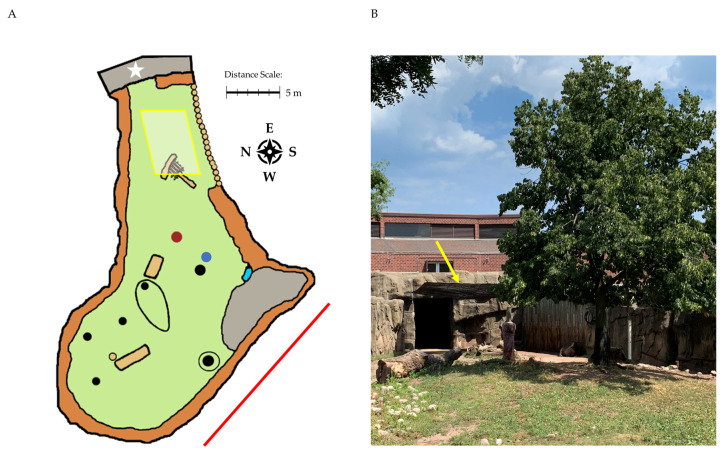
The Sichuan takin enclosure at Lincoln Park Zoo: (**A**) map showing features of the enclosure; (**B**) a photo showing the shade sail modification installed during the study. The position of the added shade sail is indicated in (**A**) with a yellow rectangle and in (**B**) with a yellow arrow. Also shown in (**A**) are a scratching post (red dot), hanging feeder (blue dot), trees (black dots), logs (tan rectangles), rock boundaries (black circles), visitor viewing area (red line), and access to indoor areas (white star).

In the second part of this study, the behavioral response of three Sichuan takin were considered. This group included two female takin and one male takin that had been housed together for over 3 years. Ages at the start of the study were 13.8 years (female 1), 11.0 years. (female 2), and 12.2 years. (male). The male takin was much larger than the females, weighing 503 kg compared to 240 kg and 288 kg for female 1 and female 2. The takin were housed in an outdoor yard (takin 1) that featured a scratching post, hanging feeder, trees, and deadfall logs (Figure 1A). In addition, a shade sail (Cosner Manufacturer, Lake Wales, FL) was installed during the study (Figure 1B). The shade sail measured 6 × 9 m and provided 12% light transmission (88% block). The shade sail was hung across a narrow portion in the rear of the enclosure leading to indoor holding areas. This location was chosen based on a lack of shade and space use in this region (see below). A sloped wall in the front of the enclosure allowed for open air viewing of the takin by zoo visitors.

### 2.3. Part 1 Data Collection: Shade Assessments 

The presence of shade was manually indicated on printed maps. Using pen and paper, observers stood near the enclosure and physically drew boundaries of shaded areas on the maps corresponding with the locations of shade present in the enclosure. To document shade conditions across the year and throughout the day, shade data were collected for each enclosure on one day per month, at three times per day: Morning (10–11 a.m.), midday (1–2 p.m.), and afternoon (4–5 p.m.). Data were collected opportunistically during the last 2 weeks of each month any day that the sunshine was significant enough to create clearly defined shadows. Data were collected from March 2018 through November 2019. Shade data were not collected December through January due to a lack of adequately sunny days and early sunset during winter. 

Shade data were converted to digital signal by using a 20 × 20 grid overlay and coding each square of the grid as “shade”, “sun”, or “not available” in Microsoft Excel. A grid square equal to or greater than 50% shaded was coded as “shade” and less than 50% shaded was coded as “sun”. Grid squares that were less than 50% within the borders of the enclosure or areas within the enclosure inaccessible to the animals were coded as “not available”. 

### 2.4. Part 2 Data Collection: Space Use Monitoring

Animal space use data were collected by volunteers as part of an ongoing zoo-wide behavioral monitoring program using the ZooMonitor application [37] on handheld tablet devices. Animal locations in the enclosures were recorded during 10-min observation sessions using interval sampling with 1-min intervals [38]. Volunteers were previously tested to ensure a minimum of 85% inter-observer reliability to be eligible to collect space use data for each enclosure. Space use data were collected by plotting the animals’ locations on a digital map in the ZooMonitor app, which converts these locations to coordinates based on a 600 × 600 coordinate point system [39]. Observers also indicated whether the animal was “in shade” (75% or more of the animal’s body in shade), “in partial shade” (25–75% of the animal’s body in shade), “in sun” (less than 25% of animal’s body in shade), or “not applicable” (when cloudy skies temporarily occluded all sun from the enclosures). At the start of each observation session, observers recorded the temperature using the Weather Channel iOS app (temperature readings from this service had been previously determined to be generally consistent with local temperatures experienced at the zoo).

Behavioral data on Sichuan takin (*n* = 3) were extracted for this study from the aforementioned behavioral monitoring project dataset. For comparison with shade mapping data and evaluation of the shade sail addition, space use data were extracted from before the shade sail addition (“pre-shade installation period”, June and July 2018; *n* = 46 sessions, 460 min of observation) and after shade sail installation (“post-shade installation period”, June and July 2019: *n* = 73 sessions, 726 min of observation). For analysis of the shade use across temperatures, data from September 2018 to March 2020 were extracted (mean observation totals per individual: 562 sessions and 5592 min). A small number of sessions (approximately 1%) were ended before 10 min due to logistical challenges; data from incomplete sessions were included in this analysis. This study was approved by the Lincoln Park Zoo Research Committee, the governing body for all animal research at the institution (No. 2018-019).

### 2.5. Data Analysis

Shade mapping data were summarized as the percent of shade availability by dividing the number of grid squares on the map marked as “shade” by the total number of grid squares for a given enclosure excluding “not available” grid squares. The percent of shade availability was first calculated for each time of the day within a month and then averaged across months for three seasons: Spring (March to May), summer (June to August), and fall (September to November).

To understand variability in shade availability, we considered the influence of season and time of day using a mixed effects model from the nlme package in the R statistical software [40,41]. Season, time of day, and their interaction were included as fixed effects in the model. A random effect (intercept) was specified for exhibit to account for repeated-measures sampling and address potential pseudoreplication.

Behavioral space use data on Sichuan takin were assessed in the form of heat maps (i.e., density plots) to evaluate locations preferences. Heat maps are visualizations that represent the density of data points using color, with red locations indicating frequently used areas and green locations representing less commonly used areas. Heat maps were generated using the MS Excel 3-D Map Visualization from space use coordinate data recorded using the ZooMonitor app. Heat maps were compared to shade maps to characterize shade availability in the areas the animals frequented the most. Heat maps were generated using data from June/July in 2018 and 2019 to visually compare the space use changes before and after installation of a shade sail in the takin enclosure. 

Behavioral shade use data scored as “in partial shade” was less frequently scored and, given observer challenges in easily distinguishing this from “in shade”, these categories were combined for analysis and hereafter referred to as “in shade”. These data were summarized as the proportion of time in shade by dividing the number of intervals with “in shade” recorded by the sum of “in shade” and “in sun” intervals recorded. In addition, observation sessions with 5 or more “not applicable” intervals (i.e., more than 50% of observation) were excluded to ensure only observation sessions during sunny days, when time in the sun or shade reflected a meaningful choice by the animals, were considered.

A logistic regression analysis was conducted to examine shade use across the full range of temperatures experienced by the takin during the year and identify the approximate temperatures at which the takin transitioned to spending more time in the shade than the sun. A logistic regression model was chosen as the distribution of data was strongly bimodal, with the proportion of shade use being 0 or 1 for 80% of the observation sessions. For analysis, observations in which the proportion of time in shade was greater than 0.5 were categorized as 1, and observations with less than 0.5 proportion of time in shade were categorized as 0. Given the limited sample size precluding population-level inferences and the importance of considering individual responses when evaluating welfare, shade use data were analyzed separately for each individual. The model was fit with temperature as a single predictor variable. Individual logistic regression models were used to calculate the cutoff temperature values for when an individual began to spend more time in the shade than sun. Logistic regression models were also generated in R.

## 3. Results

### 3.1. Part 1: Assessing Zoo-Wide Shade Availability

**Figure 2:**
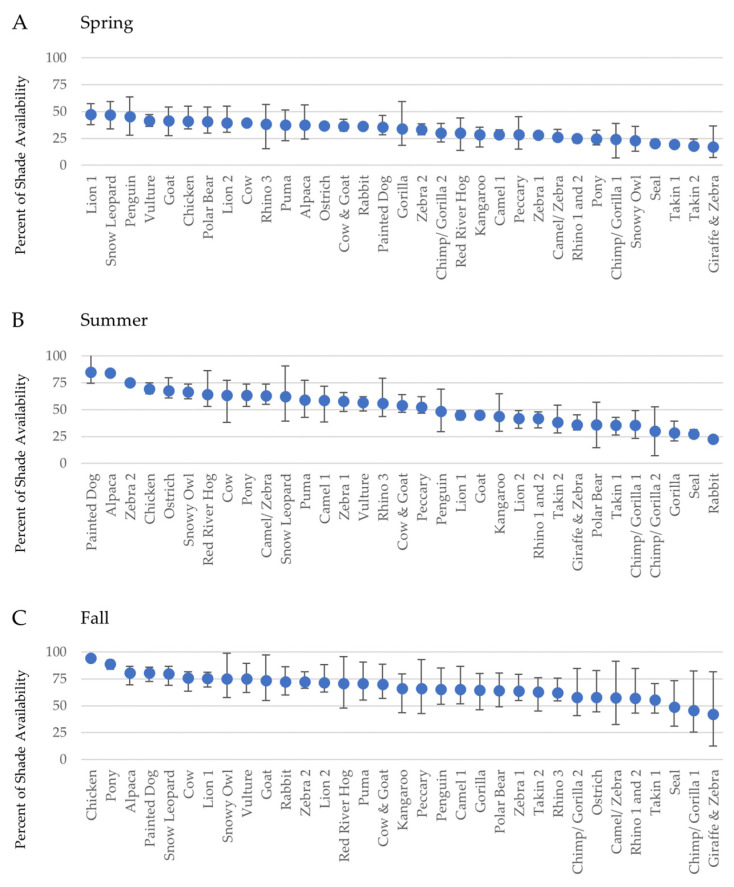
The average percent of shade availability in outdoor enclosures at Lincoln Park Zoo across the year. Error bars represent the minimum and maximum amount of shade available across three daily timepoints when shade was estimated (i.e., morning, midday, afternoon). Enclosures are arranged along the *x*-axis from most to least shade availability to demonstrate between-enclosure variability in shade availability; note the change in order between panels.

**Figure 3:**
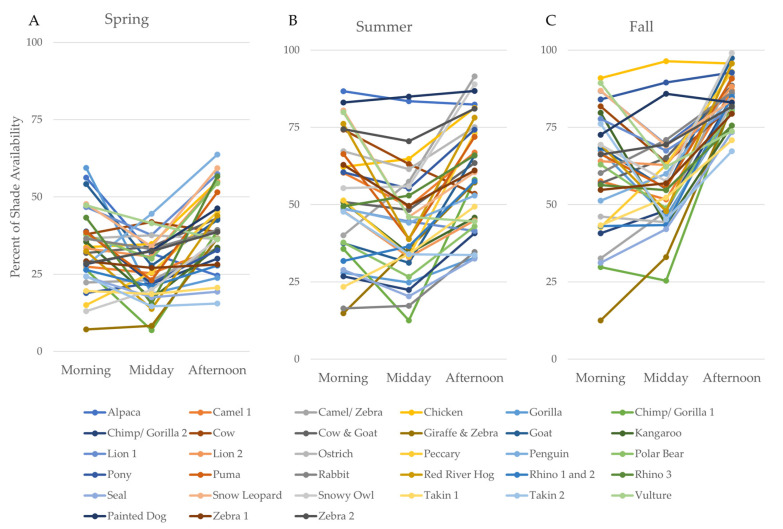
The average percent of shade availability in outdoor enclosures at Lincoln Park Zoo throughout the day for each season.

Shade availability was found to vary widely across enclosures, both by season (Figure 2) and time of day (Figure 3). This was most pronounced in summer, where the overall range of shade availability between enclosures was 62.2%, representing the difference between the enclosure with the least shade (rabbit, 22.8%) to the enclosure with the most shade (painted dog, 84.9%).

Using a generalized linear mixed model to control for enclosure differences, season (F(2256) = 197.4, *p* < 0.001), time of day (F(2256) = 59.8, *p* < 0.001), and their interaction (F(4256) = 5.0, *p* = 0.007) were all found to be significant predictors of shade availability (Table 2). As expected, the percent of shade availability increased through the year (Figure 2; Spring: $\bar{X}$ = 32.8, SD = 8.4; Summer: $\bar{X}$ = 52.0, SD = 16.2; Fall: $\bar{X}$ = 67.6, SD = 11.4). 

For most enclosures, shade varied across the day, with the least shade being available during the middle of the day (Figure 3, see Table 3 for summary statistics). Several enclosures deviated from this pattern and showed consistently high shade availability across the day: Alpaca (summer), painted dog (summer and fall), chicken (fall), and pony (fall). These enclosures had numerous trees throughout the enclosure, or nearby in the case of the chicken enclosure. Although the model identified an overall significant interaction between season and time of day, this effect was primarily attributable to the consistently high shade availability across enclosures during the fall afternoon time period, which was atypical of the wide variation in shade availability seen during other time periods (Figure 3C).

### 3.2. Part 2: Case Study: Shade Use by a Group of Sichuan Takin

**Figure 4:**
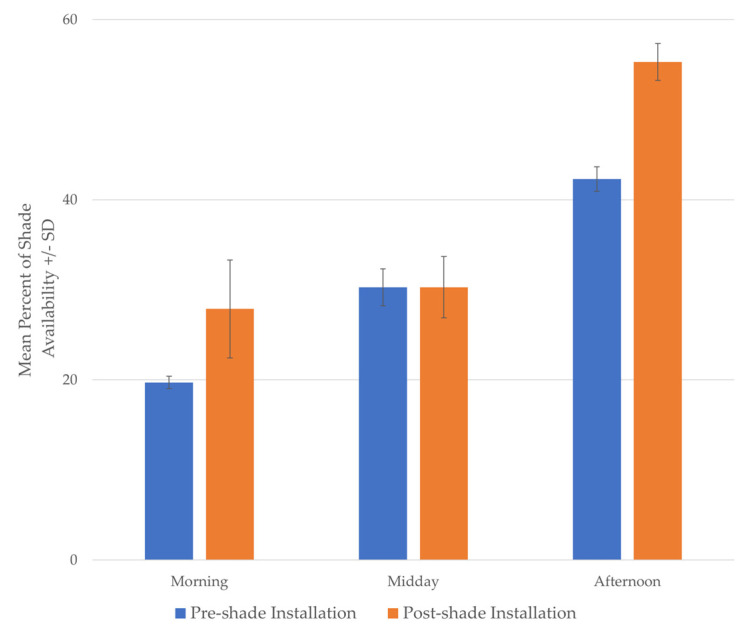
The mean percent of shade availability in the Sichuan takin enclosure at Lincoln Park Zoo before and after installation of a shade sail over a portion of the enclosure. To allow for comparisons across years, shade availability data is restricted for the months of June and July. Error bars represent the standard deviation above and below the mean.

Shade assessments from part 1 indicated limited shade availability for the Sichuan takin at Lincoln Park Zoo compared to other enclosures. To minimize the potential of heat stress on the takin, data obtained from part 1 was used to inform the location of shade sails installed in these enclosures. Data are presented for the takin 1 enclosure to demonstrate an accessible approach for evaluating enclosure modifications. In takin 1, the shade sail was erected across a narrow portion in the rear of the enclosure leading to indoor holding areas (Figure 3). The overall percent of shade availability throughout the day increased in takin 1 after the addition of the shade sail (pre-shade installation: mean = 30.8%, SD = 11.3; post-shade installation: mean = 37.8%, SD = 15.2). This increase was specifically evident during the morning and afternoon time periods; shade availability at midday remained unchanged (Figure 4).

**Figure 5:**
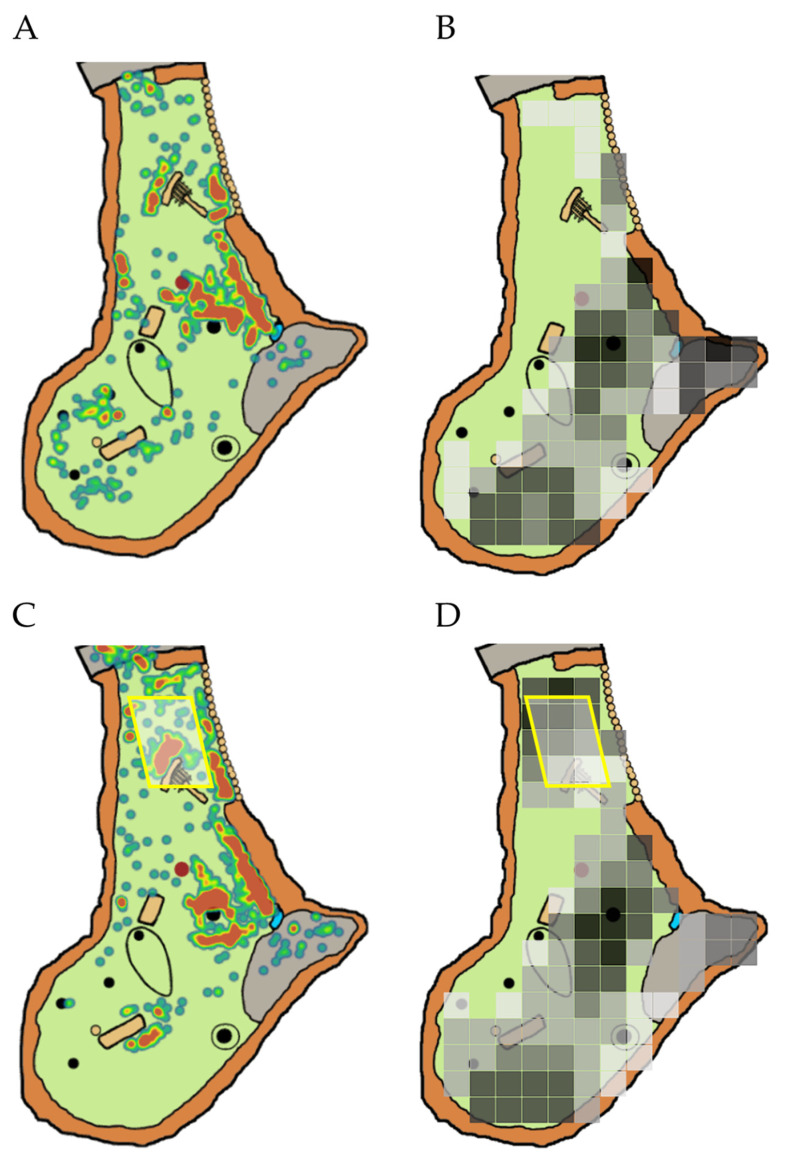
Map visualizations showing space use by three Sichuan takin (**A**) before and (**C**) after installation of a shade sail (indicated by the addition of a yellow rectangle in (**C**,**D**)) and shade availability in the enclosure (**B**) before and (**D**) after shade sail installation. Space use and shade availability are restricted to the months of June and July to allow comparisons across years. Minor changes in shade availability in the front of the enclosure (**B**,**D**) likely reflect differences in tree growth and maintenance between the two study years.

Visual inspection of the heat maps generated by the behavioral data from the pre-installation period indicated the three Sichuan takin displayed preferences for specific areas of the enclosure during the summer months (Figure 5A). These areas included space alongside the right wall of the enclosure and near a central tree, both locations that frequently offered shade (Figure 5B). Data from the post-installation period revealed use of the area under the shade sail as well (Figure 5C,D). However, the previous preference for shaded areas against the right wall of the enclosure and under a central tree remained.

**Figure 6:**
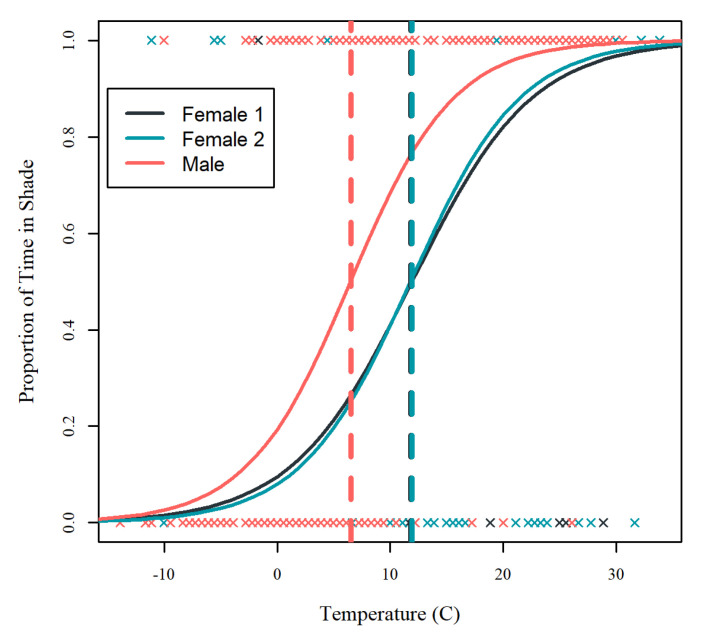
Logistic regression curves of shade use by temperature for three Sichuan takin. Observations with the proportion of shade greater than 0.5 or less than 0.5 were categorized as 1 or 0 for logistic analysis. Solid lines represent the individual logistic regression curves, dashed lines represent the 50% cutoff value when individuals used shade more than sun, and X symbols represent individual data points used for analysis.

A logistic regression analysis was conducted to examine shade use across the full range of temperatures experienced by the takin during the year and identify the approximate temperatures at which the takin transitioned to spending more time in the shade than the sun (Table 4). All three takin showed a similar pattern of minimal shade use under cold temperatures and almost exclusive use of shaded areas under high temperatures (Figure 6). The temperature threshold of when the takin began to spend more time in the shade than the sun (i.e., 50% shade use) was similar for both female takin (female 1: 11.8 degrees °C, female 2: 11.9 degrees °C). In contrast, the male takin showed a much lower threshold for shade use (male: 6.5 degrees °C).

## 4. Discussion

Animal welfare is influenced by the environment in which animals live. By creating enclosures that promote thermal comfort, zoos can increase the likelihood that animals will experience positive animal welfare. Here, we demonstrated a novel approach to quantifying the availability of shade, a dynamic environmental feature that is likely to impact the comfort of several species housed in outdoor zoo environments. We then focused in on one species, takin, and demonstrated how decisions about enclosure modifications can be made based on systematic quantification of shade and behavioral data, and evaluated the impact of that modification on subsequent behavior.

In the present study, we evaluated the availability of shade across several outdoor enclosures of a zoo that experiences seasonal variability in temperatures and sun exposure. We did so by devising a systematic method for observers to score the presence or absence of shade using a simple pen-and-paper approach. After transforming these data to quantify the percent of shade availability in an enclosure, we demonstrated that the percent of shade available varies greatly across enclosures, seasons, and time of day. Focusing in on the warmest months during the summer, when shade is most valuable to many species housed outdoors, we see that the percent of shade available across enclosures ranged from 25% to 80%. Variability emerges from enclosure characteristics, such as tree coverage, shade from adjacent or nearby buildings and walls, enclosure features, and the orientation of the exhibit. We chose to standardize shade availability by the size of the enclosure and report percentages to allow for comparison across enclosures but emphasize that absolute shade availability could be calculated with a similar approach. In some cases, the absolute amount of shade available may be more important in discussions of potential enclosure changes than the relative amount of shade.

Whether considering relative or absolute shade availability, it remains important to consider the species’ natural history, the thermal choices available in enclosures, and the number of animals in enclosures and how they use the space to provide a comprehensive understanding of how welfare is impacted by the amount of shade available. While there is certainly no target amount of shade that can be generalized across species and individuals, ensuring shade areas are utilized and large enough to allow all animals to engage in natural behaviors are likely important for promoting positive welfare. Here, consideration of the natural history of Sichuan takin led us to generate predictions about a need for more shade to provide cooling options in warmer months, given the species’ natural habitat is cooler than the summer temperatures typical in Chicago, IL. In addition, considering the space use data of individual takin at Lincoln Park Zoo, we saw that animals may have been seeking out the limited shade available in their enclosure in warmer months, leading to the addition of shade structures. Although this case study focused on the impact of shade on Sichuan takin, discussions of shade availability across zoo enclosures are underway to prioritize additional enclosures for modification and study.

Shade cloth sails represent an inexpensive option for animal managers to increase shade in zoo enclosures. In this study, we found a shade sail installed over a portion of the Sichuan takin enclosure increased the available shade in the space by roughly 10%. Unfortunately, this benefit was not observed during midday shade mapping when temperatures would have been the highest, and shade would be most important. It should be noted that additional shade from the shade sail was still present at midday, but the overhead position of the sun at this time of day minimized the shade sail’s impact. Shade was only scored in three to four grid squares of the enclosure area under the sails at this time point and the current methods were likely too broad to detect this minimal change in shade availability. Based on these findings, we recommend shade modifications should be as large as possible for the given species and evaluated at midday, at minimum, to ensure adequate shade is available during this high priority time period. Overall, the shade sails did increase the shade available to the takin and heat map visualizations indicated the takin used these areas more frequently after installation of the shade sails, along with the shade that was previously cast within their enclosure.

Using simple behavioral monitoring of shade use by individual takin, we identified a gradual transition in shade use as temperatures changed, likely reflecting the shift from sun-bathing behaviors observed under colder temperatures to shade-seeking behaviors under higher temperatures. Preferences for high sun exposure have been observed in wild takin during winter months [42] and, although shade-seeking behaviors have not been studied in takin, they are well-documented in cattle [28,29,30,43,44]. Highlighting the importance of shade for large bovids, a study that experimentally offered cows a choice between standing in shade versus lying in areas without shade after a prolonged period of forced standing found cows preferred to continue standing in shade over lying in the sun [44]. The temperatures in this study that takin began to predominantly use shade (>10 °C) were much lower than has been reported in dairy cows (>20 °C) [29,30]. Although this may reflect the natural history of takin and adaptations to colder climates, the use of app-based weather sampling in the current study and lack of detailed local temperature measurements makes these comparisons speculative.

In monitoring shade use, we noted individual differences between the takin, with both females showing a higher temperature threshold for switching to predominant shade use than the male takin (approximately a 5 °C difference). Although the male takin was socially dominant to the females and may have restricted their access to shade, this is unlikely given the distribution of shade in the enclosure. It is possible these differences may reflect the difference in body size between the male and females, as the male was nearly twice the weight of the females. Given the allometric scaling of body size in surface area-to-volume, larger individuals may be more susceptible to heat stress through increased heat production and reduced evaporation [9,45,46,47,48]. In a study on takin behavior in a managed environment, Powell et al. noted that male takin were observed to rest more in summer than female takin, suggesting larger male takin may face a greater thermal load and potentially increased risk of heat stress compared to smaller female takin [49]. Considering body size effects in future studies with larger sample sizes will allow us to test this hypothesis directly.

This study presents approachable methods to evaluating the thermal environment of zoos as a tool to promote positive animal welfare. At the broadest level, simple shade mapping with pen and paper can highlight enclosures with the most and least shade and stimulate discussions for prioritizing future detailed investigations. These shade maps can then be combined with assessments of space use, such as is available through the ZooMonitor app, to provide an understanding of animal preferences for environmental resources. More fine-grained analysis of behavioral thermoregulation is possible by documenting sun/shade use of animals across different temperatures that may describe an individual’s thermal comfort zone. While this study focused on the percent of shade availability, given the similar size of enclosures and importance for comparison across enclosures, future studies with a broader range of enclosure sizes should also consider the absolute size of shaded areas.

Incorporating temperature-dependent shade usage into behavioral observation protocols can help inform future enclosure design as well. Using data generated from the approaches described in this study, Lincoln Park Zoo has implemented a number of temperature considerations into the design of a new lion enclosure. This new enclosure was designed with a priority for shaded areas throughout the space and multiple active cooling features will be available to the lions. Kopje rocks are planned in areas with high sun exposure to provide sun-bathing opportunities during colder months. We hope these design considerations will lead to increased thermal comfort by the lions, and by extension, promote positive welfare. As the opportunity to impact welfare is much greater during the design of new enclosures than when retrofitting older spaces, we encourage others to proactively implement accessible low-cost methods as described in this study that can guide future data-driven enclosure designs.

## 5. Conclusions

In this study, we present simple, accessible methods for documenting and evaluating shade availability in zoo enclosures. Shade was found to be a highly dynamic resource for animals, varying widely both during the day and across seasons. Unfortunately, shade availability was lowest during midday periods across seasons, when shade was likely most valuable for behavioral thermoregulation. Addition of a shade sail to the takin enclosure did increase shade availability and influenced space use of the animals. The thermal environment is likely a key component of comfort for zoo animals and should be considered when designing enclosures and evaluating welfare.

## Figures and Tables

**Table 1:** Outdoor enclosures at Lincoln Park Zoo with shade assessments in this study. Domesticated species are presented as “forms” as per Zeller and Göttert [36].

Enclosure Name	Species	No. of Animals	Enclosure Size (m^2^)
Alpaca	Alpaca (*Vicugna vicugna* f. pacos)	1	554.4
Camel	Bactrian Camel (*Camelus bactrianus*)	3	1034.66
Camel/Zebra	Bactrian Camel (*C. bactrianus*), Grevy’s Zebra (*Equus grevyi*)	1, 1	652.47
Chicken	Domestic Chicken (*Gallus gallus* f. domestica)	15	87.77
Cow	Domestic Cattle (*Bos primigenius* f. taurus)	3	584.23
Cow and Goat *	Domestic Cattle (*B. primigenius* f. taurus), Domestic Goat (*Capra aegagrus* f. hircus)	1, 1	295.19
Goat	Domestic Goat (*C. aegagrus* f. hircus)	7	94.7
Kangaroo	Red Kangaroo (*Macropus rufus*)	3	378.67
Lion 2	African Lion (*Panthera leo*)	3	632.99
Lion 1	African Lion (*P. leo*)	3	657.1
Ostrich	Ostrich (*Struthio camelus*)	2	664.75
Peccary	Chacoan Peccary (*Catagonus wagneri*)	3	458.83
Penguin	African Penguin (*Spheniscus demersus*)	17	82.23
Polar Bear	Polar Bear (*Ursus maritimus*)	2	637.82
Pony	Pony (*Equus ferus* f. caballus)	3	500.3
Puma	Puma (*Puma concolor*)	1	199.48
Rabbit	Flemish Giant Rabbit (*Oryctolagus cuniculus* f. domestica)	2	21.25
Gorilla	Western Lowland Gorilla (*Gorilla gorilla gorilla*)	5, 4–7	1001.73
Chimp/Gorilla 1	Chimpanzee (*Pan. troglodytes*), Western Lowland Gorilla (*G. gorilla gorilla*)	5, 4–7	453.74
Chimp/Gorilla 2	Chimpanzee (*P. troglodytes*), Western Lowland Gorilla (*G. gorilla gorilla*)	5, 4–7	614.05
Rhino 1 and 2	Eastern Black Rhinoceros (*Diceros bicornis michaeli*)	2	1140.38
Rhino 3	Eastern Black Rhinoceros (*D. bicornis michaeli*)	1	492.37
Red River Hog	Red River Hog (*Potamochoerus porcus*)	2	265.86
Giraffe and Zebra *	Giraffe (*Giraffa camelopardalis rothschildi*), Plains Zebra (*Equus quagga*)	2, 2	1112.41
Seal *	Grey Seal (*Halichoerus grypus*), Harbor Seal (*Phoca vitulina concolor*)	1, 1	459.83
Snow Leopard	Snow Leopard (*Panthera uncia*)	1	190.22
Snowy Owl	Snowy Owl (*Budo scandiacus*)	2	78.74
Takin 1	Sichuan Takin (*Budorcas taxicolor tibetana*)	3	504.88
Takin 2	Sichuan Takin (*B. taxicolor tibetana*)	1	542.88
Vulture and Stork *	Cinereous Vulture (*Aegypius monachus*), White Stork (*Ciconia ciconia*)	2, 2	291.41
Painted Dog	African Painted Dog (*Lycaon pictus*)	2	347.21
Zebra 1	Grevy’s Zebra (*E. grevyi*)	3	1551.65
Zebra 2	Grevy’s Zebra (*E. grevyi*)	1	881.44

Asterisks (*) denote mixed-species enclosures. Forward slashes (/) denote enclosures that alternately housed different species with no cohabitation.

**Table 2:** Summary of the generalized linear mixed model predicting shade availability by season, time of day, and their interaction (denoted with ×). Significant model predictors are indicated in bold. The reference category for season is spring, the reference category for time of day is morning.

Predictor	Estimate	CI		SE	*p*
**Intercept**	32.93	27.72	38.14	2.6	<0.0001
**Season: Summer**	18.78	12.80	24.77	3.0	<0.0001
**Season: Fall**	28.26	22.28	34.25	3.0	<0.0001
**Time of Day: Midday**	−6.76	−12.74	−0.78	3.0	0.027
**Time of Day: Afternoon**	6.42	0.43	12.40	3.0	0.036
Summer × Midday	−0.98	−9.44	7.48	4.3	0.82
Fall × Midday	2.95	−5.51	11.42	4.3	0.49
Summer × Afternoon	2.21	−6.26	10.67	4.3	0.61
**Fall × Afternoon**	16.66	8.20	25.12	4.3	0.0001

CI: confidence interval; SE: standard error; *p*: probability value (α = 0.05).

**Table 3:** Average percent of shade availability across enclosures by season and time of day.

Season	Time Period	Mean	Min	Max	Range	SD
Spring	Overall	32.8	17.4	47.2	29.9	8.45
	Morning	32.9	7.14	59.4	52.3	12.2
	Midday	26.2	6.86	44.6	37.7	9.61
	Afternoon	39.3	15.5	63.8	48.2	12.4
Summer	Overall	52.0	22.8	84.9	62.2	16.2
	Morning	51.7	14.9	86.7	71.8	19.9
	Midday	44.0	12.5	84.9	72.4	17.6
	Afternoon	60.3	32.5	91.5	59.0	17.6
Fall	Overall	67.6	42.4	94.3	52.0	11.4
	Morning	61.2	12.5	90.9	78.4	19.4
	Midday	57.4	25.4	96.4	71.0	15.2
	Afternoon	84.3	67.2	99.0	31.8	7.67

SD: standard deviation.

**Table 4:** Individual logistic regression models of shade use by three Sichuan takin.

Model	Coefficient	Estimate	SE	z	*p*
Female 1	Intercept	−2.44	0.30	−8.15	<0.001
	Temperature	0.21	0.021	9.72	<0.001
Female 2	Intercept	−2.25	0.28	−7.98	<0.001
	Temperature	0.19	0.019	9.81	<0.001
Male	Intercept	−1.42	0.26	−5.56	<0.001
	Temperature	0.21	0.027	8.24	<0.001

SE: standard error of the coefficient; *p*: probability value (α = 0.05)
